# Differences in T cell cytotoxicity and cell death mechanisms between progressive multifocal leukoencephalopathy, herpes simplex virus encephalitis and cytomegalovirus encephalitis

**DOI:** 10.1007/s00401-016-1642-1

**Published:** 2016-11-05

**Authors:** Susanne Laukoter, Helmut Rauschka, Anna R. Tröscher, Ulrike Köck, Etsuji Saji, Kurt Jellinger, Hans Lassmann, Jan Bauer

**Affiliations:** 1 Department of Neuroimmunology, Center for Brain Research, Medical University of Vienna, Spitalgasse 4, 1090 Vienna, Austria; 2 Department of Neurology, Sozialmedizinisches Zentrum Ost-Donauspital, Vienna, Austria; 3 Karl Landsteiner Institut für neuroimmunologische und neurodegenerative Erkrankungen, Donauspital, Vienna, Austria; 4 Department of Neurology, Brain Research Institute, Niigata University, Niigata, Japan; 5 Institute of Clinical Neurobiology, Alberichgasse 5, 1150 Vienna, Austria

**Keywords:** Virus encephalitis, Progressive multifocal leucoencephalopathy, Cytomegalovirus, Herpes simplex virus, Cell death pathways, Caspase-mediated, Parthanatos

## Abstract

During the appearance of human immunodeficiency virus infection in the 1980 and the 1990s, progressive multifocal leukoencephalopathy (PML), a viral encephalitis induced by the JC virus, was the leading opportunistic brain infection. As a result of the use of modern immunomodulatory compounds such as Natalizumab and Rituximab, the number of patients with PML is once again increasing. Despite the presence of PML over decades, little is known regarding the mechanisms leading to death of infected cells and the role the immune system plays in this process. Here we compared the presence of inflammatory T cells and the targeting of infected cells by cytotoxic T cells in PML, herpes simplex virus encephalitis (HSVE) and cytomegalovirus encephalitis (CMVE). In addition, we analyzed cell death mechanisms in infected cells in these encephalitides. Our results show that large numbers of inflammatory cytotoxic T cells are present in PML lesions. Whereas in HSVE and CMVE, single or multiple appositions of CD8^+^ or granzyme-B^+^ T cells to infected cells are found, in PML such appositions are significantly less apparent. Analysis of apoptotic pathways by markers such as activated caspase-3, caspase-6, poly(ADP-ribose) polymerase-1 (PARP-1) and apoptosis-inducing factor (AIF) showed upregulation of caspase-3 and loss of caspase-6 from mitochondria in CMVE and HSVE infected cells. Infected oligodendrocytes in PML did not upregulate activated caspase-3 but instead showed translocation of PARP-1 from nucleus to cytoplasm and AIF from mitochondria to nucleus. These findings suggest that in HSVE and CMVE, cells die by caspase-mediated apoptosis induced by cytotoxic T cells. In PML, on the other hand, infected cells are not eliminated by the immune system but seem to die by virus-induced PARP and AIF translocation in a type of cell death defined as parthanatos.

**Electronic supplementary material:**

The online version of this article (doi:10.1007/s00401-016-1642-1) contains supplementary material, which is available to authorized users.

## Introduction

Progressive multifocal leukoencephalopathy (PML) is a demyelinating and often fatal disease caused by infection of oligodendrocytes and astrocytes in human brain [50]. It is thought to be initiated by immunosuppression-associated reactivation of latent JC virus and was first seen in immunosuppressed cancer patients and organ transplant recipients [2]. PML reached epidemic proportions as a major complication in acquired immune deficiency syndrome (AIDS) patients [9, 37]. In recent years, PML has been observed in patients treated with immunomodulatory compounds such as Natalizumab [26, 27, 29, 43] and Rituximab [17]. Generally, it is assumed that infected oligodendrocytes are eliminated by cytotoxic T lymphocytes. Indeed, JC-specific T cells can be detected in the blood of PML patients [31] and such cytotoxic CD8^+^ T cells have been found to enter the central nervous system (CNS) where they come into contact with JC-infected cells [48]. Nevertheless, at present there is no clear in situ evidence that these cytotoxic T cells are actively engaged in the elimination of infected cells.

Surprisingly little is known about the cause of oligodendrocyte cell death in PML. Some evidence of apoptosis of oligodendrocytes was shown in 2002 [41]. Others however demonstrated that infected oligodendrocytes start to express the anti-apoptotic factor surviving in order to delay apoptosis [40]. Since the first descriptions of programmed cell death mechanisms there has been a tendency to use only two categories; either cells die by apoptosis or by necrosis. At present it has become clear that there are many different pathways in which a large variety of molecules play a distinct role [24]. Besides the caspase-dependent apoptosis pathways, there is a caspase-independent pathway which involves the DNA damage-responsive enzyme poly(ADP-ribose) polymerase-1 (PARP-1). At the molecular level, expression of PARP-1 leads to formation and release of poly(ADP-ribose) (PAR) from the nucleus. In mitochondria, PAR induces release of apoptosis-inducing factor (AIF). AIF redirects from the mitochondria towards the nucleus and mediates chromatinolysis, resulting in so-called regulated necrosis [24]. This cell death pathway has been designated as parthanatos and has been suggested to play a role in a variety of pathophysiological conditions such as stroke and diabetes [18].

Here we analyzed cytotoxic T cell mechanisms and cell death pathways in (AIDS-associated) viral encephalitides PML and cytomegalovirus encephalitis (CMVE) as well as herpes simplex virus encephalitis (HSVE). Our findings suggest that in CMVE and HSVE, cells are eliminated by the immune system via a caspase-mediated pathway. In PML, infected oligodendrocytes, rather than being eliminated by cytotoxic T lymphocytes, undergo parthanatos possibly induced by the enhanced cellular activity involved in viral replication.

## Materials and methods

### Patients

This study was performed on paraffin-embedded, formalin-fixed archival material that was collected between 1982 and 1998 in the Department of Neurology at the Hospital Lainz in Vienna, Austria. The samples consisted of eight cases of AIDS-associated PML, seven cases of AIDS-associated CMVE, five cases of HSVE and five control (no neurological disease) brains. The histopathological diagnosis of these cases was established by an experienced neuropathologist (KJ). Demographic data of the individual patients are presented in Table 1.

**Table 1:** Demographic data of the patients

Sample number	Disease	Age	Male/female	Disease duration (week)	Sample area	Cause of death
12–93	PML (AIDS-assoc.)	45	Male	118	Lenticular nucleus	Pulmonary edema
47–93	PML (AIDS-assoc.)	42	Male	20	Pons	Pneumonia
97–93	PML (AIDS-assoc.)	50	Male	20	Parietal lobe right	Pulmonary edema
103–93	PML (AIDS-assoc.)	39	Male	15	Parietal lobe left	Acute respiratory insufficiency
278–93	PML (AIDS-assoc.)	29	Male	19	Parietal lobe left	Cachexia
291–93	PML (AIDS-assoc.)	49	Female	5	Parietal lobe right	Pneumonia
358–93	PML (AIDS-assoc.)	39	Male	27	Parietal lobe left	Pneumonia
359–93	PML (AIDS-assoc.)	34	Male	<18	Pons	Pneumonia
330–82	HSV	9	Female	1.5	Hippocampus	Acute respiratory insufficiency
67–83	HSV	40	Male	3.5	Hippocampus	Necrotic hemorrhagic pneumonia
204–83	HSV	61	Female	1.5	Hippocampus	Pneumonia
529–84	HSV	37	Male	2.3	Hippocampus	Pneumonia
237–91	HSV	76	Male	3.15	Hippocampus	Pneumonia
364–92	CMV (AIDS-assoc.)	40	Male	unknown	Parahippocampal region	Acute cardiovascular failure
455–92	CMV (AIDS-assoc.)	48	Male	<5	Parahippocampal region	Pneumonia, sepsis
559–92	CMV (AIDS-assoc.)	33	Male	20	Cerebellum	Pneumonia
234–93	CMV (AIDS-assoc.)	28	Male	11	Medulla oblongata	Pneumonia, sepsis
430–93	CMV (AIDS-assoc.)	35	Male	15	Medulla oblongata	Pneumonia
62–94	CMV (AIDS-assoc.)	32	Male	>4	Medulla oblongata	Pneumonia, sepsis
118–94	CMV (AIDS-assoc.)	55	Male	9	Hippocampus	Pneumonia
Control brains	–	50 (37–83)	3 Female 2 Male	–	Cortex	Non-neurological

### Histopathology and immunohistochemistry

4 μm sections of paraffin-embedded specimens were routinely stained with hematoxylin and eosin (H&E) and luxol fast blue/periodic acid-Schiff (LFB-PAS) for myelin. Immunohistochemical stainings were performed according to previously published protocols [5, 11] using primary antibodies against inflammatory T cells [CD3, CD8, CD4 and Granzyme-B (GrB)] and other inflammatory cells such as CD20^+^ B cells and CD138^+^ plasma cells. For CD3, CD8, CD4 and GrB stainings, biotinylated tyramine enhancement was used as described previously [10]. For analysis of cell death pathways we used antibodies against activated caspase-3 (CM1), caspase-6, Poly(ADP-ribose) (PAR) and apoptosis-inducing factor (AIF). For detection of virus-infected cells we used monoclonal Pab2003 [12] and monoclonal and polyclonal anti-SV40 [15], an antibody against CMV antigens and antibodies recognizing HSV-1. Light microscopical double-stainings for viral proteins and GrB or cell death markers (caspase-3) were done with an alkaline-phosphatase system (development with Fast Blue) and a peroxidase system (development with 3,3′-Diaminobenzidine (DAB) or amino ethyl carbazole (AEC) as described in detail [7]. Details on the individual antibodies are listed in Supplementary Table 1 (online resource 1).

### Confocal laser fluorescence microscopy

Fluorescence immunohistochemistry was performed on paraffin sections as previously described in detail [7]. For confocal fluorescent double-labeling or triple labeling with primary antibodies from different species, antibodies were applied simultaneously at 4 °C overnight. After washing with DAKO washing buffer (DakoCytomation, Glostrup, Denmark), secondary antibodies consisting of donkey-anti-mouse Cy3 or donkey-anti-mouse Dylight 546 (Jackson ImmunoResearch, 1:200), biotinylated donkey-anti-rabbit (Amersham Pharmacia Biotech; 1:200) and (in case of triple labeling) Cy5-conjugated donkey-anti-goat, were applied simultaneously for 1 h at room temperature, followed by application of streptavidin-Cy2 (Jackson ImmunoResearch; 1:75) for 1 h at room temperature. Fluorescent preparations were then stained with 4′,6′-diamidino-2-phenylindole (DAPI, Sigma) or TO-PRO-3 (Thermo Scientific, Waltham, MA, USA) as nuclear counterstain, embedded and examined using a confocal laser scan microscope (Leica SP5, Leica Mannheim, Germany) equipped with lasers for 504, 488, 543 and 633 nm excitation. Scanning for DAPI (504 nm), Cy2 (488 nm), Cy3 (543 nm), Cy5 (633 nm) or TO-PRO-3 (633 nm) was performed sequentially to rule out fluorescence bleed through.

### Quantification of cells

Quantification of CD3^+^, CD8^+^, CD4^+^, GrB^+^, CD20^+^ B cells, CD138^+^ plasma cells and virus-infected SV40^+^, HSV^+^ or CMV^+^ cells was performed in 5.8 mm^2^ of tissue regions exhibiting infected cells and inflammation. Typically, in PML, such areas were on the active border of large confluent chronic lesions or in small active lesions. In CMVE cases, infected cells were mostly found paraventricular (four cases) in nuclei in medulla oblongata (three cases) or in the cerebellar molecular layer (one case). In order to quantitate appositions of CD8^+^ or GrB^+^ cells with virus-infected cells we used light microscopically double-labeled sections as described above. In each case we screened 500 infected cells for the presence of single (one CD8^+^ or GrB^+^ cell attached to a virus-infected cell) or multiple (two or more CD8^+^ or GrB^+^ cells in direct contact with an infected cell) appositions. CD8^+^ cells were assessed at 200-fold magnification while GrB^+^ cells were assessed at a 400-fold magnification to be able to see the small granular signal.

Quantification of TPPP/p25+ oligodendrocytes in white matter of control brains, PML, HSVE and CMVE was performed using an ocular morphometric grid covering an area of 0.25 mm^2^ at 200-fold magnification in 2 mm^2^ of tissue regions exhibiting infected cells and inflammation (lesions) and in 2 mm of the surrounding (normal appearing) white matter.

Quantification of the percentage of virus-infected cells with caspase-3 immunoreactivity or with nuclear AIF reactivity was performed on light microscopically double-labeled sections. In each case we analyzed 500 infected cells at a 400-fold magnification.

### Statistics

Statistical analysis was performed with Graphpad Prism 6.0. For metric data, a two-sided Mann–Whitney *U* test; and for categorical data, a two-sided chi square or Fisher exact test was used as applicable. A *p* value of <0.05 was considered significant.

## Results

### Basic neuropathology

#### Progressive multifocal leucoencephalopathy

**Fig. 1:**
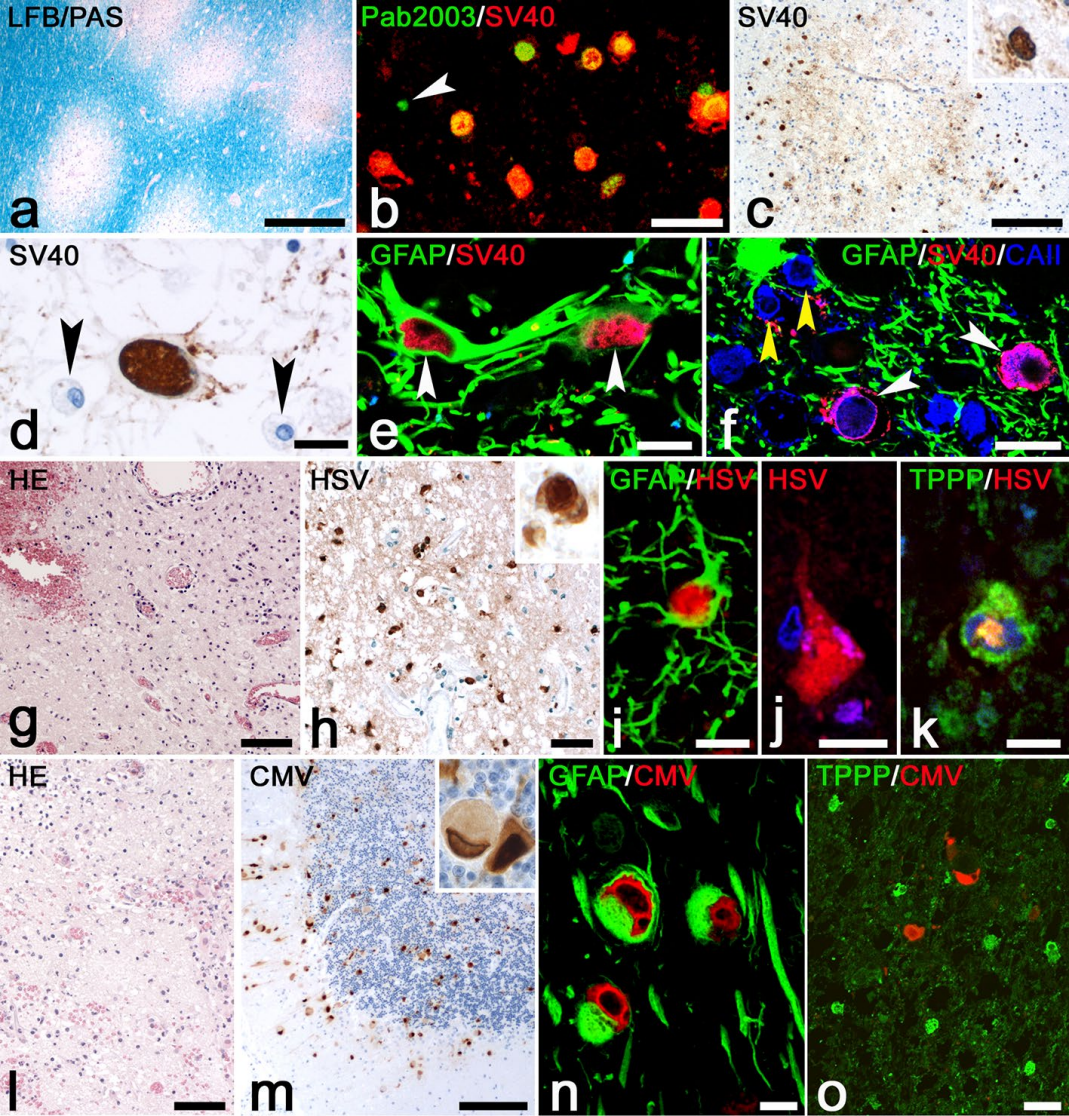
Pathology and infected cell types in PML, HSVE and CMVE. **a** LFB-PAS for myelin shows multiple demyelinated lesions in the white matter of a PML brain. Bar: 500 µm. **b** Double-staining for Pab 2003 (*green*) and SV40 (*red*) shows that most of the Pab 2003^+^ cells are also positive for SV40. The *white arrowhead* points at a single Pab2003^+^/SV40^**−**^ cell. Bar: 25 µm. **c** Staining for SV40 shows JC-infected oligodendrocytes at the border of a small demyelinated lesion. The *inset* shows an enlargement of an SV40^+^ oligodendrocyte. Bar: 200 µm. **d** Bizarre astrocyte stained for SV40 in the center of a large demyelinated lesion. The *arrowheads* point at macrophages. Bar: 20 µm. **e** Staining for GFAP (*green*) and SV40 (*red*) shows infected astrocytes in a PML lesion. Bar: 5 µm. **f** Confocal image of an area at the border of a demyelinating lesion in PML. The staining shows SV40^+^ (*red*)/CAII^+^ (*blue*) infected oligodendrocytes (*white arrowheads*) in between non-infected astrocytes stained for GFAP (*green*, *white arrow*). The *yellow arrowheads* point at two non-infected oligodendrocytes. Bar: 10 µm. **g** HE stain in HSVE showing severe hemorrhage and moderate inflammation. Bar: 200 µm. **h** Staining for HSV-1 shows large numbers of infected cells in an HSVE lesion. *Inset* shows an HSV-1^+^ cell with a nuclear inclusion. Bar: 50 µm. **i** Double-staining for GFAP (*green*) and HSV (*red*) shows an infected astrocyte in HSVE. Bar: 10 µm. **j** Here, staining for HSV shows an infected neuron. The TO-PRO-3 counterstain (*blue*) shows the nucleus of a satellite cell. Bar: 10 µm. **k** Double-staining for TPPP/p25 (*green*) and HSV (*red*) with TO-PRO-3 nuclear counterstain (*blue*) shows an infected oligodendrocyte in HSVE. Bar: 5 µm. **l** HE stain of a CMVE lesion showing inflammation and some hemorrhaging. Bar: 200 µm. **m** Staining for CMV reveals large numbers of infected cells in the cerebellum of a CMVE case. *Inset* shows an infected cell with typical owl’s eye morphology. Bar: 200 µm. **n** In CMVE many of the CMV^+^ (*red*) infected cells are GFAP^+^ astrocytes. **o** Double-staining for CMV (*red*) and TPPP/p25 (*green*) shows that in our material, lesions in CMVE do not contain infected oligodendrocytes

16 sections from 8 cases of PML were stained with LFB-PAS for detection of demyelinating lesions. Three cases contained one large lesion with a demyelinated core and with active demyelination on the border. Another two cases contained both large demyelinated lesions as well as multiple smaller demyelinating lesions. The remaining three cases had multiple smaller lesions (Fig. 1a). Quantification of oligodendrocytes in white matter showed a significant loss in and outside of PML lesions as compared to white matter of normal control brain (online resource 1, Suppl. Fig. 1). All cases were stained with Pab2003, an antibody recognizing early JCV T proteins [12]. Double-staining of Pab2003 with SV40 showed that most cells (64.5%) were double-labeled (Fig. 1b) while 6.5% were only positive for Pab2003 and 29% were only positive for SV40. Since the anti-SV40 antibody recognized more infected cells, we proceeded with this marker. In small demyelinating lesions SV40^+^ oligodendrocytes were located on the border (Fig. 1c). Unlike the larger lesions which had many (bizarre) astrocytes in the core of the lesion (Fig. 1d, e), these small lesions did not contain bizarre astrocytes. SV40^+^ oligodendrocytes, double-labeled with carbonic anhydrase II (CAII), typically had a round swollen nucleus and enlarged cytoplasm (Fig. 1f). Basic inflammation was analyzed by H&E staining showing lymphocytes in the perivascular space of blood vessels as well as infiltration in the parenchyma of the CNS. Demyelinating lesions also contained macrophages (Fig. 1d) with LFB^+^ and/or PAS^+^ myelin degradation products.

#### HSV encephalitis

Five sections from five cases showed acute haemorrhagic and subacute necrotizing lesions in both white and gray matter areas (Fig. 1g). Lesional sites in HSVE contained large numbers of inflammatory infiltrates with T lymphocytes and macrophages in meninges, the perivascular space of blood vessels and the CNS parenchyma. Numerous infected cells could be recognized by the presence of nuclear inclusions. The full amount of infected cells however only became clear after staining for HSV1, which showed HSV antigens in the nuclei and cytoplasm (Fig. 1h) of large numbers of infected astrocytes (Figs. 1i, 5b), cortical neurons (Fig. 1j) and oligodendrocytes (Fig. 1k). Like in PML, loss of oligodendrocytes in lesions was shown by quantification of stainings for TPPP/p25 (online resource 1, Suppl. Fig. 1).

#### CMV encephalitis

H&E sections showed the presence of large, often necrotic lesions with moderate infiltration of inflammatory lymphocytes (Fig. 1l). CMV-infected cells were recognized by their enlarged cytoplasm and characteristic “owl’s eye” morphology. Smaller infected cells in H&E sections were more difficult to detect but were easily found in sections stained with anti-CMV antibodies (Fig. 1m). Double-staining for CMV and GFAP showed that most of these cells were astrocytes (Fig. 1n) while oligodendrocytes were all negative for CMV (Fig. 1o). Nine sections from seven cases were studied for the presence of CMV positive cells. Almost all infected areas were found in gray matter. In four cases, groups of infected cells were found in a paraventricular position near the hippocampus. In addition, in these cases we found incidental single cells in cortical regions. Often these cells were observed in the midst of a microglial nodule. In one of these four cases a large haemorrhagic lesion was found in the hippocampus itself. In this patient we observed foci of CMV^+^ cells in cortical subpial positions. In one case, infected cells were present in small lesions of the molecular layer of the cerebellum (Fig. 1m) and in a large haemorrhagic lesion in the cerebellum. Such a large haemorrhagic lesion also was found in the medulla oblongata of a third patient. In one patient we only found single CMV^+^ cells dispersed over the medulla oblongata. Again, such cells were often found to be surrounded by microglial cells as part of a microglial nodule. Like in PML and HSVE we also quantified oligodendrocytes in the white matter but did not find any loss (online resource 1, Suppl. Fig. 1).

### T cell cytotoxicity in virus-infected brains

**Fig. 2:**
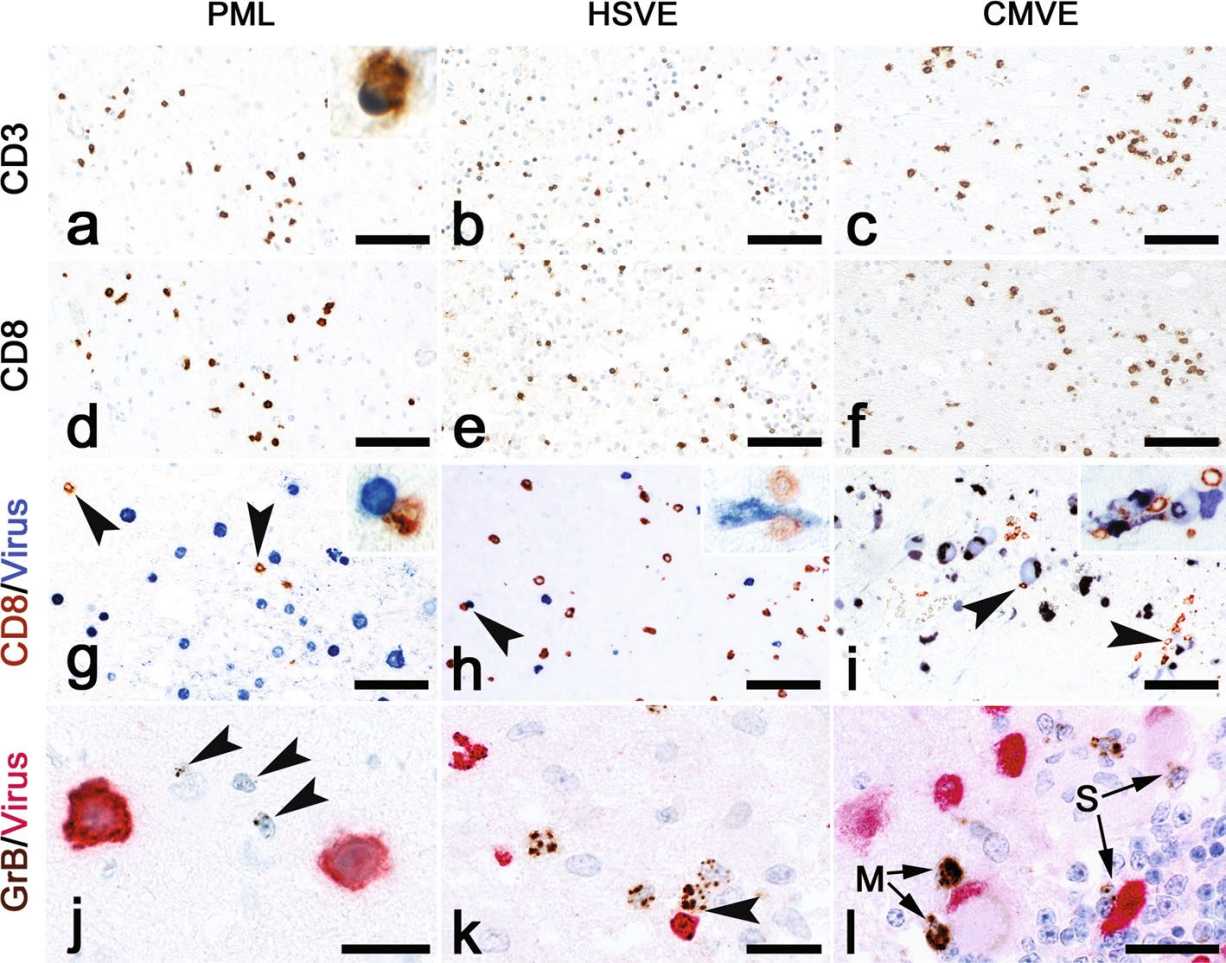
Immune mechanisms in PML, HSVE and CMV. **a–f** show identical areas of consecutive sections stained with CD3 (**a–c**) and CD8 (**d–f**) in PML, HSVE and CMVE. The stainings show that in all these encephalitides most infiltrating CD3^+^ T cells are cytotoxic CD8^+^ T cells. Bars: 100 µm Double-stainings with virus markers (*blue*) and CD8 (*brown*). **g** Few CD8^+^ T cells (*arrowheads*) can be seen between SV40^+^ cells. The *inset* shows one of the few appositions of CD8^+^ T cells to an infected oligodendrocyte. Bar: 10 µm. **h** In HSVE, CD8^+^ T cells are intermingled with small HSV^+^ cells. The *arrowhead* points at a single apposition. The *inset* shows 2 CD8^+^ T cells in apposition to an infected neuron. Bar: 10 µm **i** In CMVE many large infected cells and relatively few CD8^+^ T cells can be seen. Despite the low numbers of CD8^+^ T cells, multiple appositions (*arrowheads*) are present. The *inset* shows a cluster of CMV^+^ cells with CD8^+^ T cell appositions. Bar: 10 µm. Double-stainings with virus markers (*red*) and GrB (*brown*). **j** In PML multiple GrB^+^ lymphocytes (*arrowheads*) can be seen in between JC-infected cells. However, appositions of GrB^+^ cells to infected cells are absent. Bar: 20 µm. **k** In HSVE, a GrB^+^ T cell with polarized GrB is found in apposition to a HSV^+^ cell (*arrowhead*). Bar: 20 µm. **l** In CMVE, single (s) and multiple (m) appositions of GrB^+^ lymphocytes to CMV-infected cells can be seen. Bar: 20 µm

**Fig. 3:**
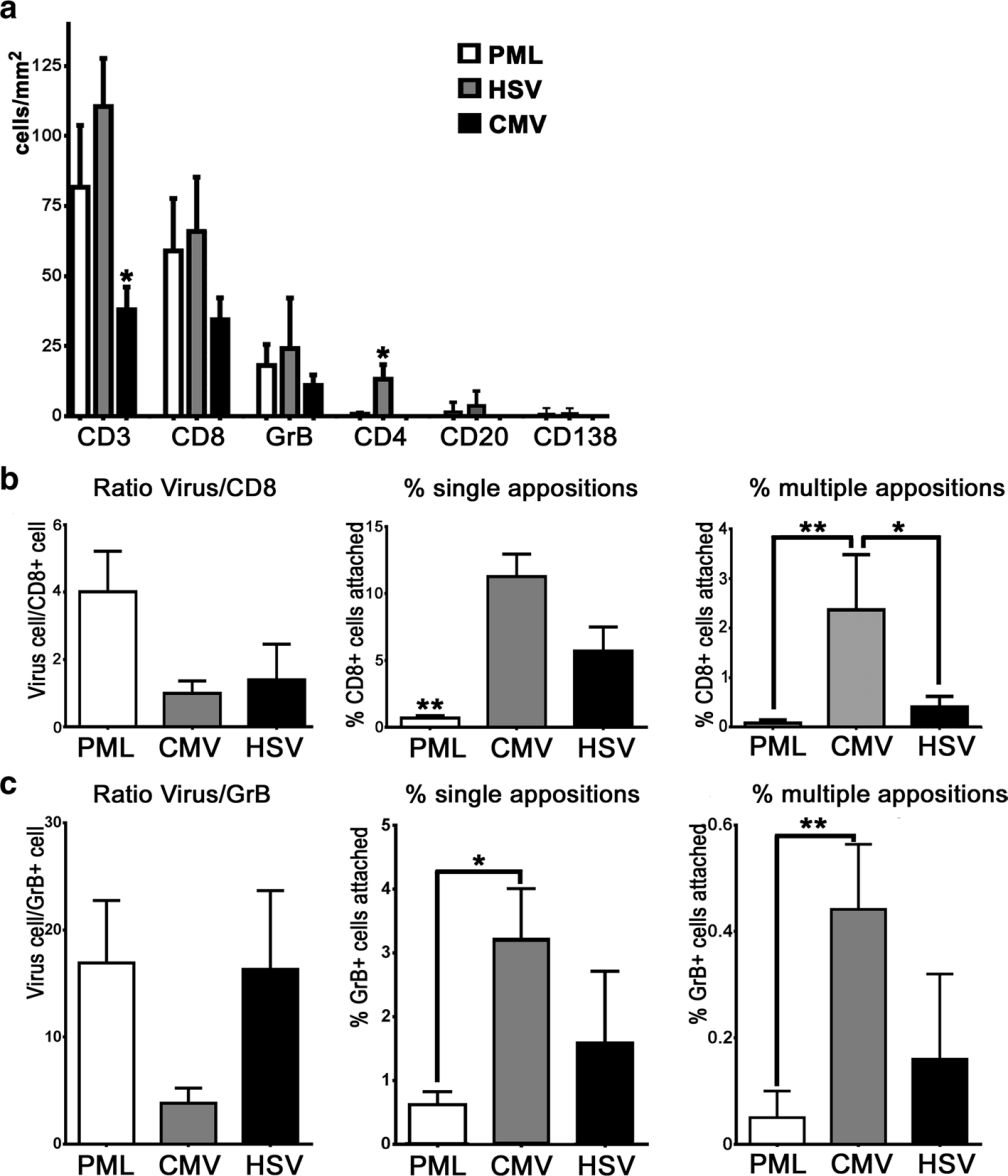
Quantitative analysis of immune cells and interactions with virus-infected cells. **a** Quantitative analysis of the numbers of CD3^+^, CD8^+^ GrB^+^, CD4^+^, CD20^+^ and CD138^+^ cells in PML, HSVE and CMVE. Average numbers of CD3^+^ T lymphocyte numbers are lower in CMV than in PML and HSVE. In addition the number of CD4^+^ T helper cells in HSVE is higher than in PML and CMVE. **b** Analysis of the ratio of virus-infected neural cells and CD8^+^ cells and the percentage of single and multiple appositions of CD8^+^ cells to virus-infected cells in PML, CMVE and HSVE. PML has significant less single appositions of CD8^+^ cells to virus-infected cells than HSVE and CMVE. CMVE has significant more multiple appositions of CD8^+^ cells to virus-infected cells than PML and HSVE. **c** Analysis of the ratio of virus-infected neural cells and GrB^+^ T cells and the percentage of single and multiple appositions of GrB^+^ T cells to virus-infected cells in PML, CMVE and HSVE. PML and CMVE differ in the number of both single and multiple appositions of GrB^+^ T cells to virus-infected cells. All numbers in (**a**–**c**) are given as average ± Standard Error of Means (SEM). *Indicates significant difference (*p* < 0.05), **indicates significant difference (*p* < 0.01)

To gain information on the role of inflammation in these encephalitides, we investigated these brains with various markers for inflammatory cells. Stainings for CD57 in PML, HSVE and CMVE demonstrated that NK cells were very rare and were mostly found in the perivascular space (online resource 1, Suppl. Fig. 2). CD20^+^ B cells and CD138^+^ plasma cells were more abundant than NK cells but less abundant than T cells (Fig. 3a). These B cells and plasma cells were mostly present in the perivascular space (Suppl. Fig. 2f and 2g). Stainings for CD3 (Fig. 2a–c) showed that T cells were present in meninges, perivascular space and deeply infiltrated in the parenchyma. Double-staining for CD8 and GrB showed that more than 95% of all GrB reactivity could be found in the CD8^+^ cells (online resource 1, Suppl. Fig. 2a). In all three forms of encephalitis we selected areas with prominent viral presence and quantified various T cell subsets in the parenchyma. Quantification revealed that in CMVE less CD3^+^ T cells were present than in PML and HSVE (Fig. 3a). In addition, quantification of CD4^+^ T Helper cells showed that their numbers were lower in CMVE and PML than in HSVE (Fig. 3a and Supp. Fig. 2D and E). The latter probably is a direct result from the elimination of CD4^+^ T cells by HIV in these AIDS-associated encephalitides. In all cases most of the infiltrating CD3^+^T cells also were CD8^+^ cells (Fig. 2d–f). In PML we found that 67% of the CD3 T cells were of the CD8 phenotype. In HSVE 58% of the CD3 T cells were CD8^+^, while in CMVE this was 89% (Fig. 3a). Granzyme-B^+^ T lymphocytes were much less frequent. In PML, 18% of CD3^+^ T cells were GrB^+^, while in HSVE and CMVE these cells reached 19 and 31% respectively (Fig. 3a). Previously, in Rasmussen Encephalitis and paraneoplastic encephalitis we showed the presence of CD8^+^ GrB^+^ cytotoxic T cells targeting neurons [10, 11]. Here we investigated whether targeting of virus-infected cells could be found. First, we quantified and calculated the ratio between virus-infected cells and CD8^+^ as well as the ratio between virus-infected cells and GrB^+^ cells. These ratios were about four times lower in CMVE than in PML, but, probably due to large interindividual variety, these differences were not significant (Fig. 3b, c). To reveal objective differences in the targeting of infected cells we quantified the appositions of single or multiple (2 or more) CD8^+^ (Fig. 3b) or GrB^+^ cells (Fig. 3c) to virus-infected cells. Surprisingly, we found that in PML both single (Fig. 2g) as well as multiple appositions of CD8^+^ (Figs. 2g, 3b) or GrB^+^ cells (Figs. 2j, 3c) were significantly less frequent than in CMVE. In addition, we also found that CMVE (Fig. 2i) contains more multiple CD8^+^ T cell appositions than HSVE (Figs. 2h, 3b). The frequency of single and multiple GrB^+^ appositions in HSVE were in between those of PML and CMVE (Fig. 3c). A specific step in active killing of target cells by cytotoxic T cells is polarization of the cytoplasm and upregulation and redirection of GrB granules towards the target cell as described in in vitro cytotoxicity [47]. In addition to the above-mentioned quantitative findings, we did observe polarization of GrB towards the virus-infected cell in HSVE (Fig. 2k) and CMVE (Fig. 2l) but not in PML (Fig. 2j).

### Cell death mechanisms in virus-infected cells

#### PML

**Fig. 4:**
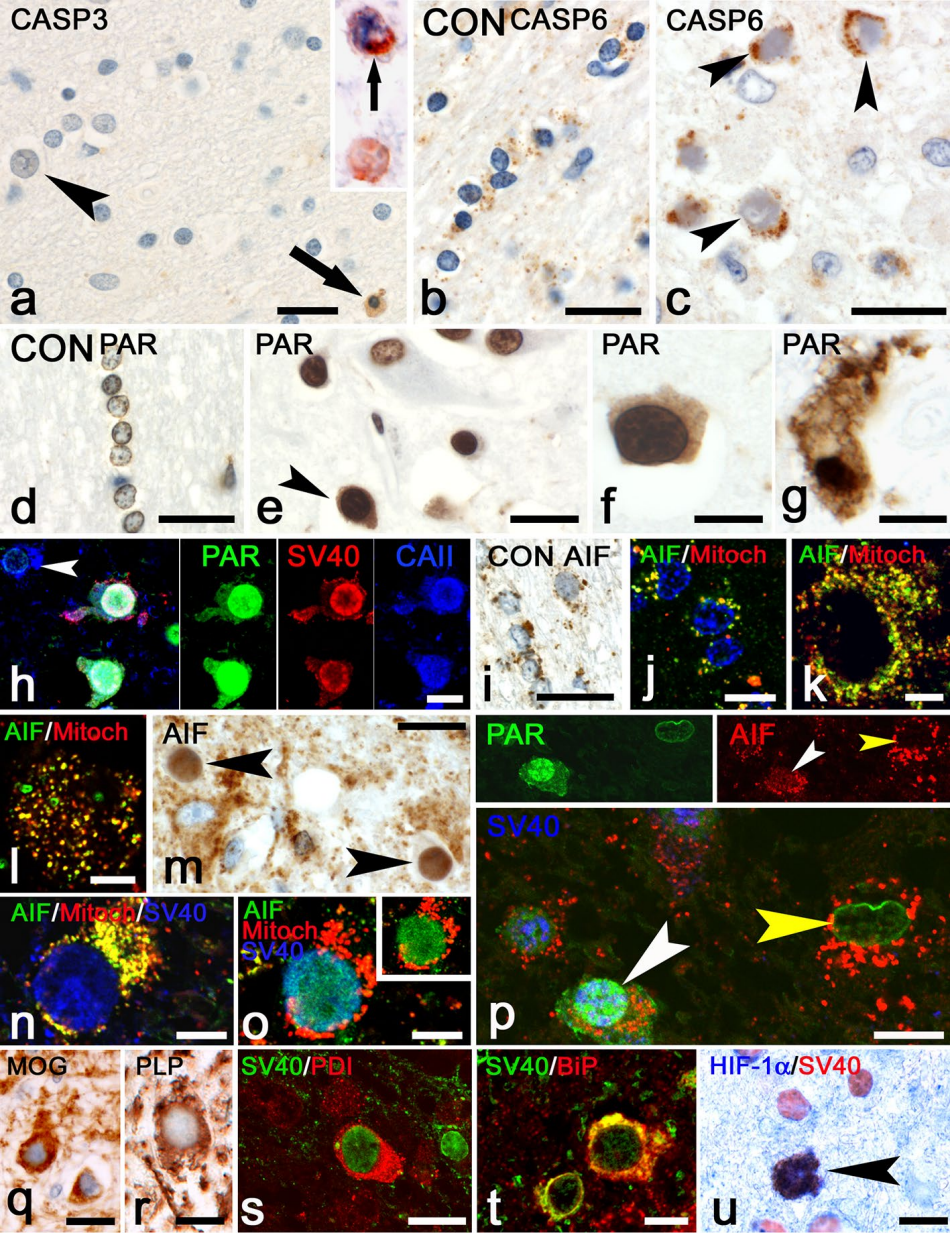
Cell death mechanisms of infected cells in PML. **a** Staining for caspase-3 shows a single apoptotic lymphocyte (*arrow*). An infected oligodendrocyte (*arrowhead*) is caspase-3 negative. Bar: 20 µm. The *inset* shows a double-staining for caspase-3 (*blue*) and CD3 (*red*) showing that the cell indicated by the *arrow* is an apoptotic T lymphocyte. **b** Staining for caspase-6 in a control brain shows a punctate mitochondrial staining in white matter oligodendrocytes. Bar: 20 µm. **c** Caspase-6 staining in PML shows upregulation of caspase-6 in infected cells (*arrowheads*) but no loss of caspase-6 from the mitochondria. Bar: 20 µm. **d** Staining for PAR in control brain. Oligodendrocytes show weak PAR reactivity in the nucleus. Bar: 20 µm. **e** PAR in PML brain. Upregulation of PAR is seen in most cells. An infected oligodendrocyte (*arrowhead*), however, reveals translocation of PAR reactivity from the nucleus to the cytoplasm. Bar: 20 µm. **f** Another example of an infected cell with translocation of PAR to the cytoplasm. Bar: 10 µm. **g** Cell with PAR immunoreactivity and vacuolation in the cytoplasm suggesting an advanced stage of degeneration. Bar: 10 µm. **h** Triple staining for PAR (*green*) SV40 (*red*) and CAII (*blue*) revealing translocation of PAR from the nucleus to the cytoplasm of an infected oligodendrocyte. The *arrowhead* points at an uninfected CAII^+^ oligodendrocyte. Bar: 10 µm. **i** Staining for AIF in a control brain. White matter oligodendrocytes show a granular immunoreactivity of AIF in mitochondria. Bar: 20 µm. Cellular localization of AIF in PML. Double-staining for AIF (*green*) and a mitochondrial marker (*Red*). **j** Here AIF is seen in mitochondria of typical uninfected oligodendrocytes. Bar: 10 µm. **k** Again, an uninfected cell (probable astrocyte) shows co-localization of AIF and anti-Mitochondria in mitochondria. **l** Also this round macrophage shows strong double-labeling indicating mitochondrial localization of AIF. **m** AIF reactivity in PML brain. Mitochondrial AIF is upregulated in many cells. Two infected cells (*arrowheads*) however show little AIF in mitochondria but strong immunoreactivity in the nucleus, suggesting translocation of AIF. Bar: 20 µm. **n** Triple staining for AIF (*green*) anti-Mitochondria (*red*) and SV40 (*blue*) shows that in this infected cell AIF is present in mitochondria. Bar: 10 µm. **o** The same triple staining shows another SV40 (*blue*) infected cell. Here, AIF (*green*) however is seen to translocate from mitochondria (*red*) to a nuclear position. The *inset* shows AIF and anti-Mitochondria in the absence of the blue (SV40) channel. Bar: 10 µm. **p** Triple staining for JC Virus (*blue*), PAR (*green*) and AIF (*red*). The infected cell indicated by the *white arrowhead* shows translocation of PAR from the nucleus to the cytoplasm. At the same time diffuse translocation of AIF to the nucleus is seen (the *inset* only shows the *green* PAR channel and the *red* AIF channel). The uninfected cell on the right indicated by the *yellow arrowhead* shows neither translocation of PAR nor from AIF. Bar: 20 µm. **q** staining for MOG shows cell bodies of infected cells. Bar: 15 µm. **r** An infected cell on the border of a demyelinated lesion also shows reactivity for PLP. Bar: 10 µm. **s** Double-staining for SV40 (*green*) and PDI (*red*) shows an infected cell with strong expression of PDI. Bar: 20 µm. **t** Such infected cells (*green*) in addition show strong reactivity for BiP/GRP78 (*red*). Bar: 10 µm. **u** Staining for HIF-1α (*blue*) shows strong nuclear upregulation in a single infected cell (SV40^+^, *red*) while other infected cells only show some weak cytoplasmic staining for HIF-1α. Bar: 20 µm

**Fig. 5:**
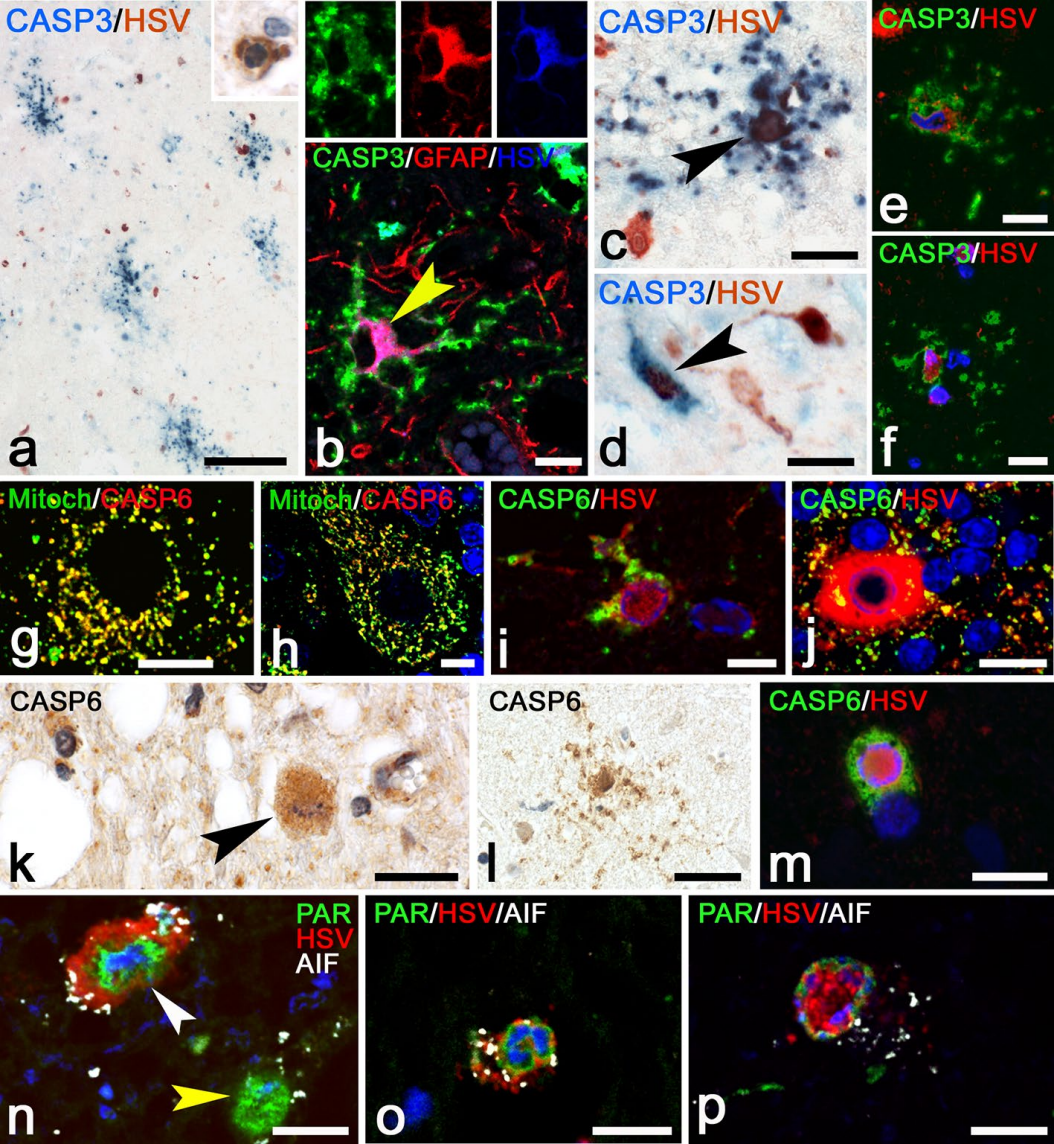
Cell death mechanisms of infected cells in HSVE. **a** Double-staining for caspase-3 (*blue*) and HSV (*red*). At this magnification, HSV is only seen in the cell body of non-apoptotic cells. The *inset* shows a single staining for caspase-3 and shows the nuclear condensation and fragmentation of this cell. Bar: 100 µm. **b** Triple staining for caspase-3 (*green*), GFAP (*red*) and HSV (*blue*) showing that the cell indicated by the *arrow* is an apoptotic astrocyte. The GFAP in the distal degenerating processes is lost. The *inset* shows this cell with separate *green*, *red* and *blue* layers. Bar: 10 µm. **c** A higher magnification of a double-staining for HSV (*red*) and caspase-3 (*blue*). Here the *arrowhead* points at an apoptotic cell which shows some HSV antigenicity in the cell body. On the left an HSV positive cell is seen. Bar: 20 µm. **d** Double-staining for HSV and caspase-3. Here the *arrowhead* points at a double-stained cell, which is a neuron as suggested by its morphology. Bar: 20 µm. The stainings in (**e,** bar 7.5 µm) and (**f**, bar: 10 µm) show caspase-3^+^ (*green*) apoptotic cells which show HSV (*red*) immunoreactivity in the cell body. Cellular localization of Caspase-6. **g** Staining for caspase-6 (*red*) and anti-Mitochondria (*green*) shows that in uninfected astrocytes caspase-6 is localized in mitochondria. Bar: 10 µm. **h** Also in this normal neuron AIF (*red*) is in a mitochondrial position. Bar: 7.5 µm. **i** Fluorescence labeling for caspase-6 (*green*) and HSV (*red*). In this infected cell most of the caspase-6 is seen in a granular mitochondrial pattern suggesting that this cell is not apoptotic. Bar: 7.5 µm. **j** This infected (probably oligodendrocyte) cell shows strong HSV reactivity (*red*) while caspase-6 (*green*) is present in few mitochondria. Bar: 10 µm. **k** Single staining for caspase-6. The *arrowhead* points at a cell with diffuse labeling of the cytoplasm and a fragmented nucleus. Bar: 20 µm. **l** Caspase-6 reactivity in an apoptotic astrocyte of which the processes show fragmentation. Bar: 20 µm. **m** Here double-labeling shows a diffuse staining for caspase-6 (*green*) and reduced HSV reactivity (*red*, only in nuclear local ization) suggesting that this cell is undergoing apoptosis. Quadruple stainings for PAR (*green*), HSV (*red*), AIF (*white*) and DAPI nuclear counterstain (*blue*). **n** The *white arrowhead* points at a non-apoptotic HSV-infected cell. PAR is present in the nucleus while AIF is located in a granular mitochondrial location. Bar: 7.5 µm. The *yellow arrowhead* points at a non-infected cell. Again PAR is found in the nucleus while AIF is located in mitochondria. **o** In this HSV^+^ cell (*red*) the nucleus (*blue*) clearly shows fragmentation. PAR (*green*) however is still present in a nuclear position while AIF (*white*) remains in the mitochondria. Bar: 7.5 µm. **p** Another infected cell with nuclear fragmentation indicating apoptosis. PAR again is present in the nucleus while AIF is present in mitochondria. In this case HSV reactivity (*red*) is reduced and only present in a nuclear position as also seen in Fig. 5e, f, m. Bar: 7.5 µm

Activated caspase-3 and caspase-6 play a central role in caspase-mediated apoptosis which can be induced by CD8^+^ cytotoxic T cells through release of GrB. We hypothesized that if cytotoxic GrB^+^ T cells eliminate virus-infected cells in the various encephalitides, we should be able to detect apoptotic virus-infected cells with upregulated activated caspase-3, or loss of caspase-6 from mitochondria [23]. Previously, we have shown that upregulation of activated caspase-3 is found in the cytoplasm in apoptotic cells, [3–5, 13]. Here, in normal control brain, activated caspase-3 upregulation was not present. In PML, staining for activated caspase-3 showed the presence of small apoptotic cells (Fig. 4a) that co-localized with CD3 (inset, Fig. 4a) and therefore were characterized as lymphocytes. Analysis of infected oligodendrocytes at the border of demyelinating lesions showed that none of these cells were reactive for caspase-3 (Fig. 4a; Table 2). In control brain (Fig. 4b) and in uninfected cells (Fig. 5g–h), caspase-6 was present in mitochondria. In PML, caspase-6 immunoreactivity was strongly increased. In infected oligodendrocytes, caspase-6 was seen in a granular mitochondrial pattern (Fig. 4c), with no detectable translocation to the cytoplasm being observed.

**Table 2:** Percentage of infected cells with caspase-3 or nuclear AIF expression

	PML	HSV	CMV
Caspase-3	0.0 ± 0.0*	0.40 ± 0.13	4.97 ± 1.77
Nuclear AIF	1.6 ± 0.40**	0.00 ± 0.00	0.00 ± 0.00

Data are given as percentage ± SEM

* *p* values: PML vs. HSV, *p* = 0.0013, PML vs. CMV, *p* = 0.02, CMV vs. HSV, *p* = 0.20

** *p* values: PML vs. HSV, *p* = 0.006, PML vs. CMV, *p* = 0.004, CMV vs. HSV, *p* > 0.99

Since oligodendrocyte death in our PML cases does not seem to result from cytotoxic T cell induced activation of the caspase-cascade, we investigated caspase-independent pathways of cell death. One of these pathways is the Poly(ADP-ribose) polymerase-1-(PARP-1) dependent cell death pathway in which poly-(ADP-ribose) (PAR) and apoptosis-inducing factor (AIF) are some of the key molecules [23]. In normal cells of control brain, weak staining for PAR was seen in the nucleus of cells (Fig. 4d). In PML, enhanced PAR reactivity in uninfected cells was observed (Fig. 4e). In addition, in some of the infected oligodendrocytes, PAR not only was present in the nucleus, but also was seen to translocate to the cytosol (Fig. 4e–h). Some of these cells showed largely degenerating cytoplasms with large vacuoles (Fig. 4g). Staining for AIF in control brain revealed the granular presence of AIF restricted to the mitochondria of normal cells (Fig. 4i). In PML brain, AIF reactivity was upregulated in the mitochondria of uninfected cells (Fig. 4j–l) as well as in mitochondria of most of the infected oligodendrocytes (Fig. 4n). In part of the infected cells, however, we observed that staining of AIF was largely absent from the mitochondria and was translocated to the nucleus (Fig. 4m, o, p). Quantification showed that AIF translocation to the nucleus was detectable in 1.6% of these cells (Table 2). Confocal fluorescence triple staining for SV40, PAR and AIF confirmed the presence of infected cells with simultaneous translocation of PAR from nucleus to cytoplasm and AIF from mitochondria to nucleus (Fig. 4p).

In normal adult brain, oligodendrocyte cell bodies do not express myelin proteins such as major oligodendrocyte glycoprotein (MOG) and proteolipid protein (PLP). During analysis of our PML tissues we however noticed that some of the infected oligodendrocytes at the border of demyelinated lesions strongly express MOG (Fig. 4q) and PLP (Fig. 4r) in the cell bodies. These findings suggest that these proteins are trapped in the endoplasmic reticulum (ER), something we also have shown previously in PLP overexpressing transgenic animals with ER stress [13]. Analysis of protein disulfide isomerase (PDI) and Binding Protein/Glucose Regulated Protein 78 (BiP/GRP78), both involved in endoplasmic reticulum stress, revealed strong upregulation in such infected oligodendrocytes (Fig. 4s, t). ER stress also has been shown to induce hypoxia inducing factor-1α (HIF-1α) [8]. Previously, we already showed the presence of HIF-1α in cases of PML [1]. Here, double-staining for HIF-1α with SV40 shows that HIF-1α is strongly upregulated in some of the infected cells (Fig. 4u).

#### HSV encephalitis

To detect apoptosis in infected cells we performed double-labeling for caspase-3 and HSV. These stainings revealed the presence of large numbers of positive cells of which, based on their morphology, most were recognized as astrocytes (Fig. 5a). Quantification of these stainings showed that on average 0.4% of infected cells were caspase-3^+^ (Table 2). The presence of infected apoptotic astrocytes was confirmed by triple labeling of HSV with caspase-3 and GFAP (Fig. 5b). Many of these apoptotic astrocytes showed degeneration of the astrocytic processes (Fig. 5b–c) as also seen previously in Rasmussen encephalitis [5]. Besides these astrocytes also some infected neurons showed caspase-3 reactivity (Fig. 5d). In many of these apoptotic cells, HSV immunostaining became less strong and HSV antigens were only present in the cell body of these cells (Fig. 5e–f). HSVE brains also were stained for caspase-6. In both uninfected (Fig. 5g–h) as well as in many HSV-infected cells (Fig. 5i–j), caspase-6 was present in mitochondria. In areas with large numbers of degenerating caspase-3^+^ cells we also found caspase-6^+^ cells with apoptotic nuclei (Fig. 5k). Interestingly, such apoptotic cells showed a diffuse cytoplasmic staining for caspase-6 and loss of mitochondrial caspase-6 (Fig. 5k). Like in caspase-3 stainings, many of these caspase-6^+^ cells were astrocytes (Fig. 5l), although also cells with oligodendrocyte morphology with cytoplasmic staining of caspase-6 (Fig. 5m) were found.

Since it was difficult to discriminate small HSV-infected cells from normal cells, we also analyzed HSVE brains by performing quadruple labeling of HSV with PAR, AIF and nuclear DAPI stain. These stainings clearly showed that in infected cells, PAR always remained in the nucleus (Fig. 5n–p). Staining for AIF in HSV-infected cells, on the other hand, never was seen outside of the mitochondria (Table 2; Fig. 5n–p). Furthermore, part of the HSV^+^ cells, again showed a diminished reactivity for HSV, present only in nucleus and cell body (Fig. 5p).

#### CMV encephalitis

**Fig. 6:**
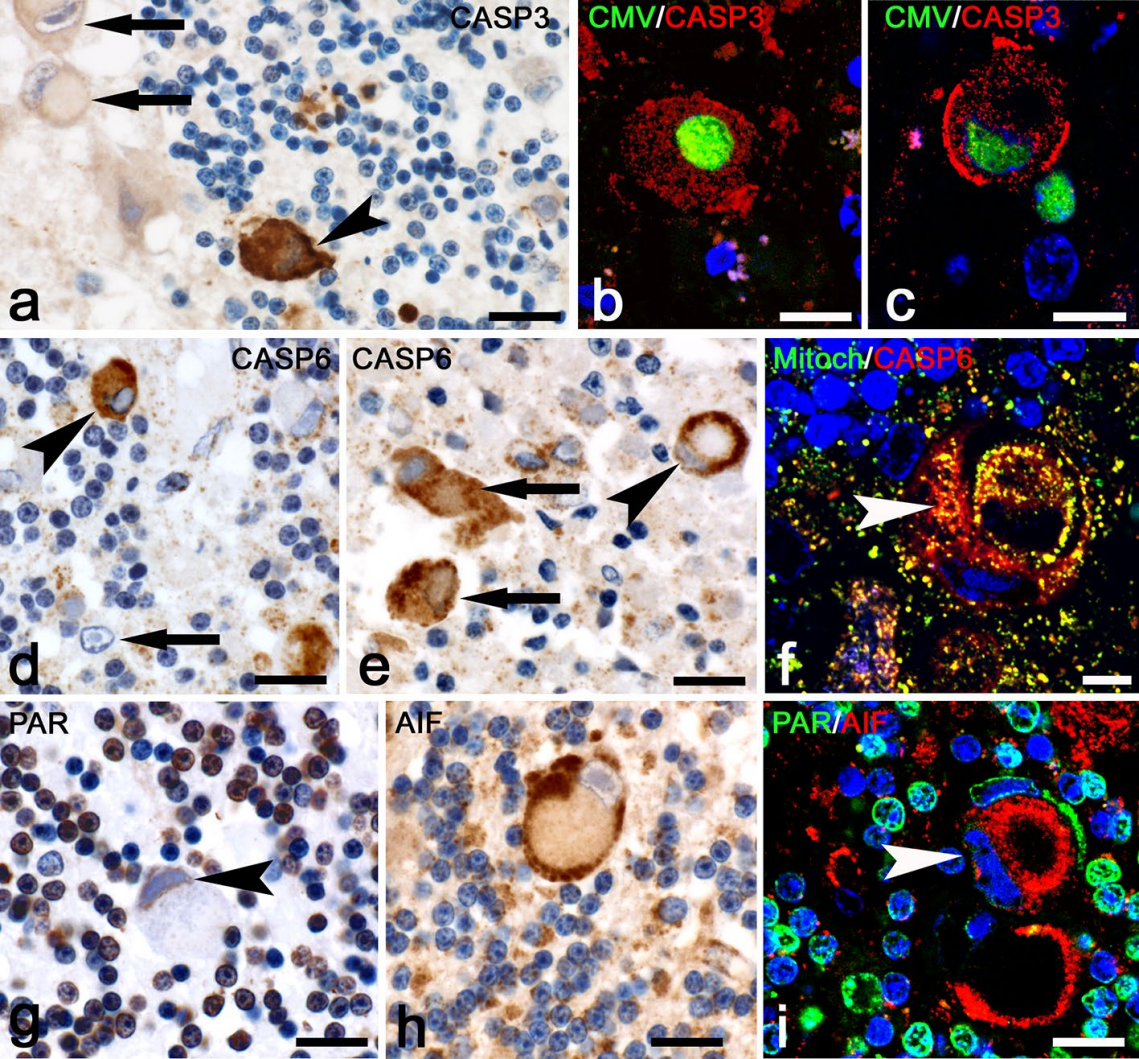
Cell death mechanisms of infected cells in CMVE. **a** Infected cell (*arrowhead*) strongly stained for activated caspase-3. The CMV-infected cells on the left upper corner (*arrows*) show no reactivity for caspase-3. Bar: 20 µm. **b**, **c** Two examples of infected cells double-stained for CMV (*green*) and upregulation of activated caspase-3 (*red*). Bar in b: 20 µm. Bar in c: 10 µm.** d**,** e** Staining for caspase-6. In **d** the *arrow* points at a CMV-infected cell with very little caspase-6^+^ mitochondria. The *arrowhead*, on the other hand, points at an infected cell with a strong cytoplasmic localization of caspase-6, suggesting apoptosis. Bar: 20 µm. **e** Here, the *arrowhead* points at an infected cell with a mitochondrial staining for caspase-6. Notice that the mitochondria in this cell are gathered close to the cell membrane. The other two infected cells (*arrows*) show both caspase-6 in a granular (mitochondrial) as well as cytoplasmic location. Bar: 20 µm. **f** Confocal fluorescence staining shows a typical CMV-infected cell with caspase-6 immunoreactivity (*red*) outside of mitochondria (*green*). Bar: 7.5 µm. **g** Staining for PAR shows immunoreactivity in the nucleus of an infected cell (*arrowhead*). In none of the infected cells did we observe PAR in a cytoplasmic location. Bar: 20 µm. **h** This staining reveals a mitochondrial localization of AIF in a large infected cell. Again, mitochondria are located closely to the cell membrane. Staining for AIF never showed any staining in the nuclei of infected cells, suggesting the absence of AIF-associated apoptotic pathways in these cells. Bar: 20 µm. **i** Fluorescence double-staining for PAR (*green*) and AIF (*red*). The *white arrowhead* points at an infected cell. Weak PAR reactivity is present in a nuclear position while AIF is present in a granular mitochondrial localization. Bar: 20 µm

In CMVE, like in PML, activated caspase-3 again could be found in lymphocytes. In addition we found strong cytoplasmic upregulation of caspase-3 in infected cells (Fig. 6a). This finding was confirmed in double-labeling studies for caspase-3 and CMV where almost 5% of CMV^+^ cells were reactive for caspase-3 (Table 2; Fig. 6b, c). In comparison to other cells, part of the CMV-infected cells only revealed little granular caspase-6 immunoreactivity (arrow Fig. 6d). Some of the infected cells with apoptotic nuclei showed strong cytoplasmic staining (arrowhead Fig. 6d) in line with caspase-mediated apoptosis. Other infected cells however revealed intense caspase-6 immunoreactivity in a granular mitochondrial pattern (Fig. 6e). Notably, unlike in uninfected cells, caspase-6^+^ mitochondria in some infected cells seemed to be located close to the cell membrane (Fig. 6e). In other CMV-infected cells, caspase-6 immunoreactivity was seen both in mitochondria as well as diffusely dispersed in the cytoplasm (arrows Fig. 6e, f). To confirm that the cell death mechanism in CMVE differs from PML, we also investigated PAR and AIF expression. Immunoreactivity for PAR in infected cells was solely present in the nucleus and never found in a cytoplasmic localization (Fig. 6g, i). In addition, much like the staining for caspase-6, mitochondrial AIF showed a granular pattern mainly located at the outer side of the cytoplasm close to the cell membrane (Fig. 6h, i). AIF reactivity in CMV-infected cells was never found to translocate to the nucleus (Table 2). Altogether, the absence of translocation of PAR and AIF, the presence of activated caspase-3 and the translocation of caspase-6 from mitochondria to cytoplasm in CMV and HSV-infected cells strongly suggest that these cells, unlike cells in PML, die by caspase-mediated apoptosis.

## Discussion

The introduction of new highly potent anti-inflammatory drugs, such as Natalizumab and Rituximab has led to an increasing incidence of PML in cancer and autoimmune disease patients [6]. In addition, variants of PML with co-existence of the immune reconstitution inflammatory syndrome (IRIS), also known as PML-IRIS, have in recent years been brought up [6, 35]. Previously, we and others have shown that in PML-IRIS the numbers of inflammatory T cells and plasma cells are increased about 50-fold as compared to PML [34, 36]. Analysis of PML and PML-IRIS cases showed that numbers of infected cells in PML-IRIS were decreased and direct apposition of cytotoxic T cells towards virus-infected cells was increased, suggesting that the reconstituted immune system in PML-IRIS indeed eliminates virus-infected cells more rigorous than in PML [33]. In that study we also analyzed apoptosis of infected cells in PML or PML-IRIS but did not detect any activated caspase-3 immunoreactive oligodendrocytes [33].

Since the question regarding the death of infected cells in PML remained open, we decided to analyze cell death pathways and inflammatory reactions in more detail. Like in our previous study [33], in PML we could not demonstrate a significant attack of JC-infected cells by cytotoxic T cells. This is in contrast with CMVE and HSVE where cytotoxic GrB^+^ T cells in apposition to virus-infected cells were more obvious. Notably, the absence of cytotoxic T cells targeting infected oligodendrocytes in PML is not because of a lack of MHC class I expression, since we have shown previously that these cells reveal strong MHC class I immunoreactivity [6]. Probably one of the reasons for a more effective elimination of virus-infected cells in CMVE compared to PML is that within lesions the ratio of GrB^+^ T cells per virus-infected cell seems higher in CMVE. In HSVE absolute numbers of CD3^+^ T cells and CD4^+^ T helper cells were much higher than in CMVE and PML. This may be related to the AIDS-associated compromised immune system. The relatively low number of appositions in HSVE probably again is a reflection of the unfavorable ratio between HSV-infected cells and GrB^+^ T cells. Overall, our results suggest that in PML the immune system is not able to contain the virus infection due to a low number of inflammatory cells relative to the number of infected oligodendrocytes.

Previously, in animal models for EAE we showed that oligodendrocytes and astrocytes undergo caspase-3-mediated apoptosis after attack by CD8^+^ cytotoxic T cells [16, 42]. In addition, in human Rasmussen Encephalitis we revealed that cells are attacked by cytotoxic T cells and undergo caspase-3 mediated apoptosis [5, 10]. These findings suggest that elimination of neural cells by cytotoxic T cells predominantly is achieved by induction of caspase-mediated apoptosis. Here, in CMVE and HSVE we indeed detected infected cells with caspase-3 reactivity in areas where infected cells were attacked by cytotoxic T cells. These findings are in line with findings by DeBiasi et al. who detected caspase-3 in infected cells in acute HSV encephalitis and congenital CMV encephalitis of mostly young children [19]. These authors at that time however could not demonstrate a role of the immune system as we did here. Our findings in CMVE and HSVE are in sharp contrast with PML, where we did not find any caspase-3 reactivity in the cytoplasm of JC-infected cells.

Despite being a central pathological feature, until now very few studies dealt with the question of oligodendrocyte death in PML. One paper favored apoptosis as the death inducing mechanism [41], whereas the other study argued that apoptosis is not involved in oligodendrocyte death since these cells are protected by the anti-apoptotic protein survivin [40]. Richardson-Burns et al. showed DNA fragmentation in oligodendrocytes by TUNEL. They however did not identify morphological changes in the nucleus, as usually seen in apoptotic cells. Moreover, caspase-3 in this study was observed in the nucleus rather than the cytoplasm. Although these findings are striking at first glance, there are some caveats which at that time were not considered. First, it has been reported that DNA viruses cause DNA double-strand breaks in host cells [25, 28, 44, 49] and thus it is conceivable that rather than apoptosis or necrosis, the TUNEL staining detects DNA double-strand breaks induced by the JC virus. Secondly, we and others have shown that apoptotic cells with nuclear condensation or the presence of apoptotic bodies show cytoplasmic rather than nuclear distribution of activated caspase-3 [3, 13, 14, 20, 45]. Moreover, nuclear caspase-3 has been suggested to play a role other than in apoptosis [38].

Instead of activated caspase-3, infected degenerating oligodendrocytes in PML expressed nuclear AIF and cytoplasmic PAR. In addition, in these cells we could show signs of ER stress and upregulation of hypoxia inducible factor-1α (HIF-1α). The translocation of PAR and AIF suggests that these cells died by a pathway designated as parthanatos [18, 23]. Interestingly, parthanatos is often linked to cell death induced by hypoxia [32]. Also in PML a role of hypoxic mechanisms has been suggested [30, 39]. Hypoxia might be induced by molecules such as nitric oxide or oxygen radicals produced by macrophages and microglia. In addition, hypoxic pathways may result from thrombotic occlusion of small inflamed vessels [21]. Another possibility, however, is that a virtual hypoxia is induced in JC-virus-infected cells indirectly. By taking over the host’s protein production machinery, viruses can induce endoplasmic reticulum (ER) stress [22]. More specifically, ER stress can be induced by so-called viroporins [22]. In JC-virus-infected cells, agnoprotein acts as such a viroporin [46]. ER stress by viroporins is induced by multiple mechanisms such as membrane remodeling, delayed glycoprotein trafficking and disruption of calcium homeostasis. The latter results in an increase in cytosolic calcium followed by calcium-dependent cell death signaling and finally cell death [22, 46]. ER stress therefore, can act as an intracellular inducer of hypoxic pathways as shown in intermittent hypoxia where ER stress induced HIF-1α activity [8].

In summary, our results show that, compared to CMVE and HSVE, interaction of GrB^+^ cytotoxic T cells with infected oligodendrocytes are much less frequent in PML lesions. Furthermore, in CMVE and HSVE, infected cells seem to die by caspase-mediated apoptotic pathways. In PML however, infected oligodendrocytes show translocation of PAR and AIF, hypoxia and ER stress which are all compatible with the parthanatos pathway of cell death. Our findings suggest that, unlike in HSVE and CMVE, in PML the immune system not adequately enough eradicates JC-infected oligodendrocytes. Instead these cells seem to die by virus-induced changes in homeostatic pathways.

## Electronic supplementary material

Below is the link to the electronic supplementary material.

Supplementary material 1 (DOCX 342 kb)

Supplementary material 2 (DOCX 2252 kb)

Supplementary material 3 (DOCX 23 kb)
