# Supplementary material for: Differences in T cell cytotoxicity and cell death mechanisms between progressive multifocal leukoencephalopathy, herpes simplex virus encephalitis and cytomegalovirus encephalitis
Source: Acta Neuropathol. 2016 Nov 5;133(4):613–27. doi: 10.1007/s00401-016-1642-1 (PMC5348553; doi:10.1007/s00401-016-1642-1)
Supplement: Supplementary file 1 — Supplementary material 1 (DOCX 342 kb) [file 401_2016_1642_MOESM1_ESM.docx]

**Supplementary Figure 1. Oligodendrocyte loss in PML, HSVE and CMVE.**


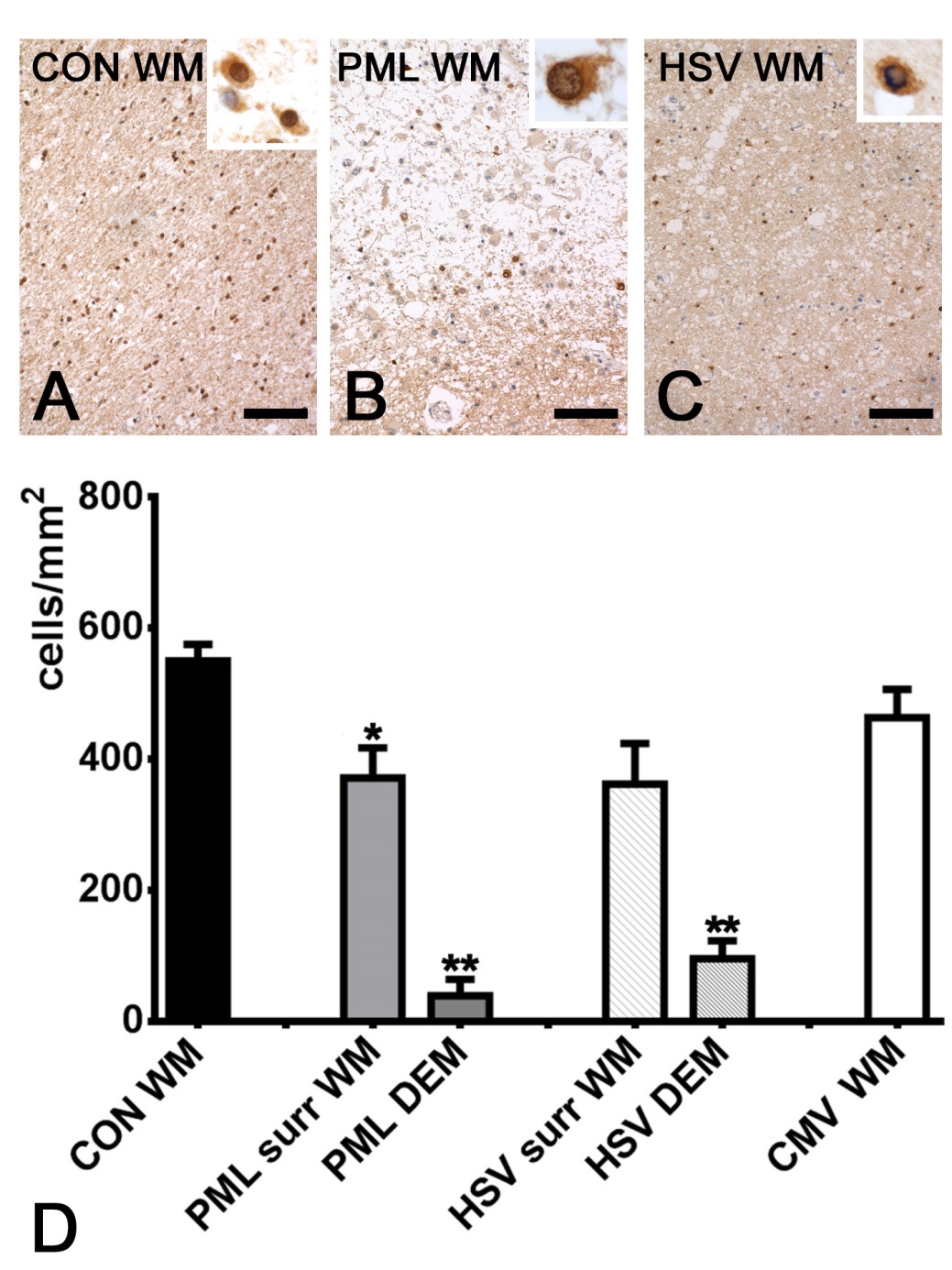


**Supplementary Figure 1. Oligodendrocyte loss in PML, HSVE and CMVE. (A-C)** Staining for TPPP/p25. (A) Staining in normal white matter (CON WM). (B) Staining in PML at the edge of a demyelinating lesion with loss of oligodendrocytes. The inset shows an infected oligodendrocyte within the lesion. (C) Staining in demyelinated WM of HSV encephalitis. The inset shows an apoptotic oligodendrocyte with condensed nucleus. Bar in A-C: 100 µm. (D) TPPP/p25^+^ oligodendrocytes were measured in white matter of control patients (CON WM), in demyelinated white matter lesions in PML and HSV encephalitis (PML DEM and HSV DEM) and in white matter surrounding demyelinated lesions (PML surr WM and HSV surr WM). In CMV encephalitis almost all infected areas where in grey matter, therefore only white matter (CMV WM) was analysed. *significantly different from CON WM (p<0,05), ** significantly different from CON WM (p<0,01).
