# Supplementary material for: Differences in T cell cytotoxicity and cell death mechanisms between progressive multifocal leukoencephalopathy, herpes simplex virus encephalitis and cytomegalovirus encephalitis
Source: Acta Neuropathol. 2016 Nov 5;133(4):613–27. doi: 10.1007/s00401-016-1642-1 (PMC5348553; doi:10.1007/s00401-016-1642-1)
Supplement: Supplementary file 2 — Supplementary material 2 (DOCX 2252 kb) [file 401_2016_1642_MOESM2_ESM.docx]

**Supplementary Figure 2**

**
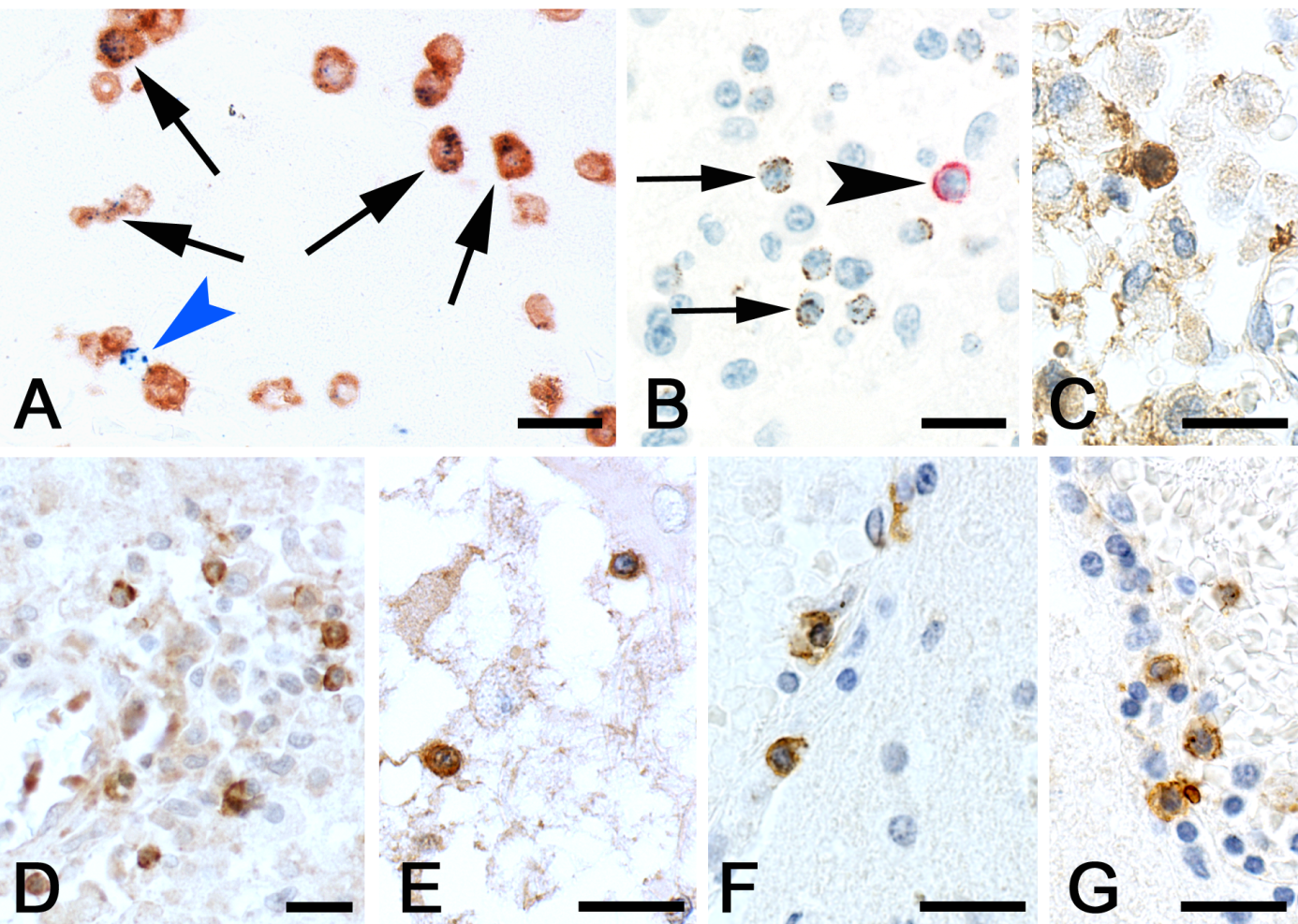
**

**Supplementary Figure 2. Immune cells in virus encephalitis.** (A) Double staining for GrB (blue) and CD8 in PML, shows that almost all GrB-reactivity is found in CD8^+^ cells (arrows). The blue arrowhead points at a single GrB^+^ CD8^-^ cell. (B) GrB (brown) and CD4 (red) in HSVE. No GrB-reactivity was found in CD4^+^ cells. (C) Staining for CD57 shows a rare Natural Killer (NK) cell in a HSVE lesion. (D) In HSVE moderate numbers of CD4^+^ T cells are present in perivascular cuffs and in the surrounding parenchyma. (E) In PML, some CD4^+^ cells are present, but are much less abundant than in HSVE. (F) Staining for CD20 in PML shows that most B cells are located in the perivascular space of a bloodvessel. (F) Also CD138^+^ plasma cells are mostly found in the perivascular space. All Bars: 20 µm
