# Supplementary material for: Differences in T cell cytotoxicity and cell death mechanisms between progressive multifocal leukoencephalopathy, herpes simplex virus encephalitis and cytomegalovirus encephalitis
Source: Acta Neuropathol. 2016 Nov 5;133(4):613–27. doi: 10.1007/s00401-016-1642-1 (PMC5348553; doi:10.1007/s00401-016-1642-1)
Supplement: Supplementary file 3 — Supplementary material 3 (DOCX 23 kb) [file 401_2016_1642_MOESM3_ESM.docx]

Supplementary Table 1: Primary antibodies used for immunohistochemistry and immunofluorescence

| **Antibody** | **Antibody type** | **Target** | **Preatreatment** | **Dilution** | **Source** |
| --- | --- | --- | --- | --- | --- |
| CD3 | Rabbit (poly AB) | T cells | steamer 60' EDTA 9.0 | 1:2000 with CSA | Labvision, Fremont, CA |
| CD8 | Mouse (mAB) | Cytotoxic T-cells | steamer 60' EDTA 9.0 | 1:250 | Dakopatts, Hamburg, Germany |
| GrB | Mouse (mAB) | Granzyme B | steamer 60' EDTA 9.0 | 1:50 or 1:1000 with CSA | Labvision, Fremont, CA |
| CD4*  CD4* | Mouse (mAB)  Mouse (mAB) | T helper cells | steamer 60' EDTA 9.0  steamer 60' EDTA 9.0 | 1:500 with CSA  1:100  with CSA | Dako (M7310)  Acris (DM119-05) |
| CD57 | Mouse (mAB) | NK cells | steamer 60'  EDTA 9.0 | 1:100 | ThermoFisher (MS-136-P) |
| CD20 | Mouse (mAB) | B cells | steamer 60'  EDTA 9.0 | 1:100 | Thermo scientific  (CD20 Ab-1) |
| SV40  SV40  SV40**  SV40**  SV40** | Rabbit (poly AB)  Rabbit (poly AB)  Mouse (mAB)  Mouse (mAB)  Mouse (mAB) | T-Antigen | steamer 60'  EDTA 9.0  steamer 60' EDTA 9.0  steamer 60' EDTA 9.0  steamer 60' EDTA 9.0  steamer 60' EDTA 9.0 | 1:5000 or  1:20000  with CSA  1:200  1:2000  with CSA  1:1000  with CSA  1:1000 | Gift Dr. Höftberger, Vienna, Austria (Budka and Shah, 1983)  Santa Cruz (sc-20800)  Oncogene (DPO2)  San Diego, CA  Merck Millipore (Pab416)  Becton Dickinson  (Pab 101) |
| Pab2003 | Mouse (mAB) | JC-Virus early T | steamer 90' EDTA 9.0 | 1:2000  with CSA | Gift from Dr. Richard Frisque (Bollag et al., 2000) |
| CMV | Mouse (mAB) | early antigen of CMV | steamer 60' EDTA 9.0 | 1:5000 | Merck Millipore  (Mab8131) |
| HSV | Rabbit (poly AB) | HSV type I and II | steamer 60' citrate | 1:1000 | Dakopatts, Hamburg, Germany |
| CM1 | Rabbit (poly AB) | activated Caspase-3 | steamer 60' citrate | 1:3000 | Becton Dickinson, San Diego, CA |
| Caspase-6 | Rabbit (poly AB) | active Caspase-6 | steamer 60' citrate | 1:500 | Abcam, Oxford, UK  (ab 52295) |
| AIF | Rabbit (poly AB) | Apoptosis inducing factor | steamer 60' citrate | 1:250 | Chemicon, Temecula, CA |
| AIF | Sheep (polyAB) | Apoptosis inducing factor | steamer 60' citrate/EDTA 9.0 | 1:1000 | R&D systems, Minneapolis, MN |
| anti-Mitochondria | Mouse (mAB) | 56 kd mitochondrial membrane protein | steamer 60' EDTA 9.0 | 1:1000 | Merck Millipore  MAB1273 |
| Bip/Grp78 | Mouse (mAB) | binding protein  78 kD glucose regulated protein | steamer 60' EDTA 9.0 | 1:500 | Transuction Laboratories Lexington, KY |
| PDI | Mouse (mAB) | Protein Disulfide Isomerase | steamer 60' EDTA 9.0 | 1:100 | Stressgen (SPA-891), Victoria, BL Canada |
| HIF-1α | Mouse (mAB) | hypoxia inducible factor-1α | steamer 60' EDTA 9.0 | 1:2000 | Novus Biologicals (NB100-131), Littleton, CO |
| PAR | Mouse (mAB) | Poly (ADP-ribose) | no pretreatment | 1:250 | Alexis Biotechnology (clone 10H),  London, UK |
| CAII | Sheep (polyAB) | Carbonic Anhydrase II | steamer 60' EDTA 9.0 | 1:1000 | The Binding site (pc076),  Birmingham, UK |
| TPPP/p25 | rat (mAB) | tubulin polymerization promoting protein TPPP/p25 | steamer 60' EDTA 9.0 | 1:500 | Gift. Dr. Kovacs, Vienna, Austria  (Kovács et al., 2007 |
| GFAP | Mouse (mAB) | Glial fibrillary acidic protein | steamer 60' citrate | 1:200 | Thermo Scientific (Ms 1376), Fremont, CA |
| GFAP | Rabbit (poly AB) | Glial fibrillary acidic protein | steamer 60' EDTA 9.0 | 1:3000 | Dakopatts, Hamburg, Germany |

## mAB = monoclonal antibody; polyAB = polyclonal antibody, CSA: Catalysed System Amplification using biotinylated tyramine (see Materials and methods). * These antibodies were mixed to imrove the sensitivity. ** These antibodies were tested but on this material were found too insensitive to use.
